# The Relationship between Economic Status, Knowledge on Dengue, Risk Perceptions and Practices

**DOI:** 10.1371/journal.pone.0081875

**Published:** 2013-12-12

**Authors:** Marta Castro, Lizet Sánchez, Dennis Pérez, Carlos Sebrango, Ziv Shkedy, Patrick Van der Stuyft

**Affiliations:** 1 Epidemiology Division, Institute of Tropical Medicine “Pedro Kouri”, Havana, Cuba; 2 Clinical Research Direction, Center of Molecular Immunology, Havana, Cuba; 3 Department of Mathematics, José Martí University, Sancti Spirítus, Cuba; 4 Center for Statistics, Hasselt University, Diepenbeek, Belgium; 5 Unit of General Epidemiology and Disease Control, Institute of Tropical Medicine, Antwerp, Belgium; 6 Department of Public Health, Ghent University, Ghent, Belgium; Alberta Provincial Laboratory for Public Health/University of Alberta, Canada

## Abstract

The reemergence of dengue as an important public health problem reflects the difficulties in sustaining vertically organized, effective, control programs and the need for community-based strategies for *Aedes aegypti* control that result in behavioral change. We aimed to disentangle the relationships between underlying determinants of dengue related practices. We conducted a cross-sectional study in 780 households in La Lisa, Havana, Cuba. A questionnaire and an observation guide were administrated to collect information on variables related to economic status, knowledge on dengue, risk perception and practices associated with *Aedes aegypti* breading sites. To test a conceptual model that hypothesized direct relationships among all these constructs, we first used Exploratory Factor Analysis with Principal Component Analysis to establish the relationship between observed variables and the underlying latent variables. Subsequently, we tested whether the observed data supported the conceptual model through Confirmatory Factor Analysis. Exploratory Factor Analysis indicated that the items measured could be reduced into five factors with an eigenvalue >1.0: Knowledge on dengue, Intradomiciliar risk practices, Peridomiciliar risk practices, Risk perception and Economic status. The proportion of the total variance in the data explained by these five factors was 74.3%. The Confirmatory Factor Analysis model differed from our hypothesized conceptual model. Only Knowledge on dengue had a significant, direct, positive, effect on Practices. There was also a direct association of Economic status with Knowledge on dengue, but not with Risk perception and Practices. Clarifying the relationship between direct and indirect determinants of dengue related practices contributes to a better understanding of the potential effect of Information Education and Communication on practices and on the reduction of *Aedes aegypti* breeding sites and provides inputs for designing a community based strategy for dengue control.

## Introduction

Dengue infections currently occur in more than 100 countries in the Asia-Pacific region, the Americas, the Middle East, and Africa [1]. The reemergence of dengue as an important public health problem reflects the difficulties in maintaining vertically organized, effective, control programs and the need to develop sustainable, integrated approaches to *Aedes aegypti* control [1], [2]. Community participation and changes in human behaviors are essential components [3], [4], [6], since programs that include information, education and communication and strengthen active participation achieve better results [5]–[8].

Dengue KAP surveys have frequently been used to describe knowledge, attitudes and practices of the community towards prevention [9]–[14]. They can identify knowledge gaps, cultural beliefs or behavioral patterns that form barriers for action, and assist in the design of promotion activities [15]–[17]. They have also been used as a tool to assess the effectiveness of health education programs and community based strategies [5]–[7], [18]–[25]. However, a review on dengue prevention indicates that there is little evidence on how information from KAP surveys was used to plan for subsequent participatory strategies [17]. Furthermore, while education campaigns have increased people’s knowledge on dengue, it remains unclear to what extent this led to behavioral change and actually reduced mosquito populations [8].

KAP studies indicate that the relationships between determinants of human behaviour with bearing on the control of *Ae. aegypti* are complex [3]. Some studies used regression models to examinate the direct relationships [6], [11]–[13], [23], [26], but the challenge is to characterize the behavioral system and to understand the direct and indirect effects of the determinants. Latent variable structural equation modeling provides a tool to address this challenge and allows for the quantification and testing of hypothesized relationships among latent and observed variables [27].

The present study was carried out as part of the formative research prior to developing a community-based strategy for dengue prevention [28], [29]. Our aim was to disentangle the relationships between Economic status, Knowledge on dengue, Risk perception and dengue related Practices.

## Materials and Methods

### Study Area and Design

The study was conducted in La Lisa municipality, Havana, Cuba. The municipality has about 30 000 inhabitants, and an area of 37.5 km^2^. The population is concentrated in residential neighborhoods with urban characteristics; at the periphery the territory is semi-urban to rural. Three amongst the seven “Consejos Populares” (CP: an intermediate governance structure between the municipal level and the “circunscripción”, the lowest level of local government that covers about 1 000 inhabitants), Versalles-Coronela, Alturas de La Lisa and Balcon Arimao are high-risk areas for dengue transmission, with House Index (number of houses with at least one container with *Ae. aegypti* larvae/100 houses examined) higher than 2% [28]. We conducted a descriptive cross-sectional survey in these CPs in November 2004. 32 circumscriptions were randomly selected and the heads (or, if absent, another adult resident) of 780 randomly selected households were interviewed.

### Underlying Conceptual Model

**Figure 1:**
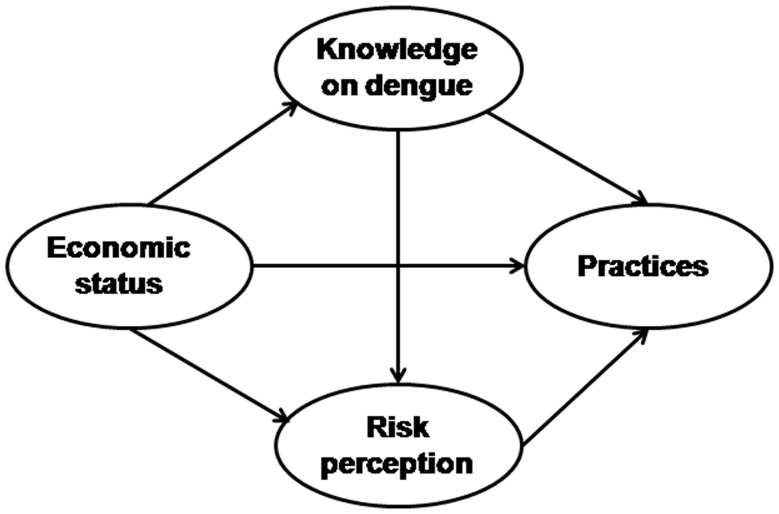
Underlying conceptual model for studying the relationships between Economic status, Knowledge on dengue, Risk perception and Practices.

Based on previous research [6], [9], [11], [23], [26], measure of Economic status, Knowledge on dengue, Risk perception and Risk practices for dengue were collected. The underlying hypothesis (Figure 1) were that better economic status is directly associated with decreased risk practices for dengue, with higher knowledge on dengue and with increased risk perception. Secondly, that knowledge is associated with a higher risk perception and better practices. Finally, that higher risk perception is associated with decreased risk practices for dengue.

### Data Collection

Trained local health volunteers used two instruments for the study: a questionnaire created and validated previously [4] and an observation guide elaborated to assess risk behavior associated with *Ae. aegypti* breading sites.

The questionnaire covered socio-demographic information (age, education); knowledge on dengue symptoms (measured as the number of correct symptoms that respondents mentioned amongst fever, headache, nausea/vomiting, muscular pain, rash or bleeding); knowledge of preventive measures (the number of measures mentioned amongst adding temephos to water storage containers, covering other useful containers, eliminating useless containers, spraying insecticide); and risk perception (the risk to contract dengue fever and whether dengue infection could be fatal, both scaled as none(1), moderate(2), or high(3)). In addition, we ascertained the respondents number of information sources on dengue (e.g. television, radio, newspaper, local leaders, doctors, nurses, local meetings). Finally, a scale was developed for owning items related to economic status. An air-conditioning system, video recorder, telephone, car or microwave were given 3 points each; a refrigerator, fan, washing machine or mixer 2 points; and a radio, sewing machine, pressure cooker or tape recorder each 1 point. All points were summed to obtain a household asset score.

The practices related to dengue that we observed were the number of water storage containers that were badly covered or in bad condition, the number of other small containers outside the house and the number of useless containers in the backyard.

### Data Analysis

Firstly, we carried out a descriptive analysis of the KAP survey. After that, an Exploratory Factor Analysis (EFA) was conducted to determine the best factor structure to represent the relationships between economic status, knowledge on dengue, risk perception and dengue related practices. A Principal Component Analysis (PCA) was applied using orthogonal rotation (Varimax). Those factors with an eigenvalue larger than 1 were retained. We tested whether the observed data supported the underlying conceptual model with a Confirmatory Factor Analysis (CFA) [30]. To perform the CFA we followed six basic steps according Hatcher [31]: 1. Define the factor model (the underlying conceptual model); 2. Collect measurements; 3. Obtain the correlation matrix; 4. Fit the model to the data (*Maximum likelihood* estimation); 5. Evaluate model adequacy (chi-square test and *goodness-of-fit* test); and 6. Compare with other models (comparing a full model with a reduced model by examining the difference between their *X^2^* statistics).

All statistics were performed with SPSS 15.0 for Windows except the CFA for which SAS PROC CALIS was used. We drew the path diagram with Graphviz 2.26.3. All p-values are two-tailed and considered statistically significant at the 5% level.

### Ethics Statement

The study was approved by the Research and Ethics Committee of the Institute Pedro Kouri, the Infectious Diseases Research Committee of the Cuban Ministry of Health and the municipal health authorities. The participants were provided with a verbal and written explanation of the objectives and procedures of the study. Written informed consent was obtained from all respondents prior to the interview. The ethic committee waived the need for informed consent from the next of kin, caretakers, or guardians on behalf of respondents aged 16 or 17. The topic explored was not considered as a personal or sensitive issue for such ages. During the investigation process, no information that could distinguish individual respondents was collected.

## Results

### Descriptive Analysis

Table 1 summarizes the characteristic of the 779/780 respondents for whom complete information was available. Approximately 50% of the respondents were between 30–59 years old and 60% had completed secondary education. They mentioned, on average, correctly 3 dengue symptoms and 4 preventive measures. 39% of the respondents thought they were not at risk to contract dengue, but more than 87% referred that dengue could be highly fatal. The mean number of badly covered water storage containers and of useless containers in the backyard was one and 5 per household, respectively. The mean household asset score was 15.

**Table 1:** Characteristics of respondents. La Lisa municipality, Havana, Cuba.

Characteristic	n = 779
Age group (years)	
16–29	12.2%
30–44	26.7%
45–59	23.5%
+59	37.6%
Education	
Primary	22.4%
Secondary	35.6%
Technical training	34.1%
University	7.9%
Mean (SD) of number of dengue symptoms known	3.09 (1.82)
Mean (SD) of number of preventive measures known	4.24 (1.39)
Mean (SD) of number of sources of information about dengue reported	5.90 (2.44)
Perception of risk to contract dengue	
High	21.1%
Moderate	39.8%
None	39.2%
Perception that dengue can be fatal (%)	
High	87.2%
Moderate	4.1%
None	8.7%
Mean (SD) of badly covered water storage containers	1.24 (1.29)
Mean (SD) of water storage containers in bad condition	0.29 (0.94)
Mean (SD) of useless containers in the backyard	5.84 (9.47)
Mean (SD) of other small containers outside household	2.74 (5.84)
Mean (DP) of household asset score	15.40 (5.81)

### Exploratory and Confirmatory Factor Analysis

EFA (Table 2) showed that the 10 items measured could be reduced into five factors with an eigenvalue >1.0: Knowledge on dengue, Intradomiciliar risk practices, Peridomiciliar risk practices, Risk perception and Economic status. The proportion of the total variance in the data explained by these five factors was 74.34%.

**Table 2:** Exploratory factor analysis: Factor loading and explained variance.

Variables	Factor 1 Knowledgeon dengue	Factor 2 Intradomiciliarrisk practices	Factor 3 Peridomiciliarrisk practices	Factor 4 Risk perception	Factor 5 Economic status
Knowledge of preventive measures	**0.79**	0.00	−0.07	−0.01	−0.09
Knowledge of dengue symptoms	**0.70**	−0.07	−0.09	0.17	0.10
Information source on dengue	**0.79**	−0.04	0.02	0.04	0.12
Water storage containers in bad condition	−0.56	**0.93**	−0.09	−0.02	−0.01
Badly covered water storage containers	−0.05	**0.93**	0.09	−0.00	−0.02
Useless containers in the backyard	−0.09	−0.01	**0.91**	0.00	−0.02
Other small containers outside the house	−0.03	0.00	**0.91**	0.03	−0.01
Perception of risk to contract dengue	0.01	−0.01	−0.06	**0.79**	0.08
Perception that dengue can be fatal	0.01	−0.01	0.09	**0.72**	0.04
Household asset score	0.09	0.03	−0.03	0.03	**0.98**
**Explained variance (%)**	**21.36**	**17.39**	**15.20**	**10.79**	**9.59**

**Figure 2:**
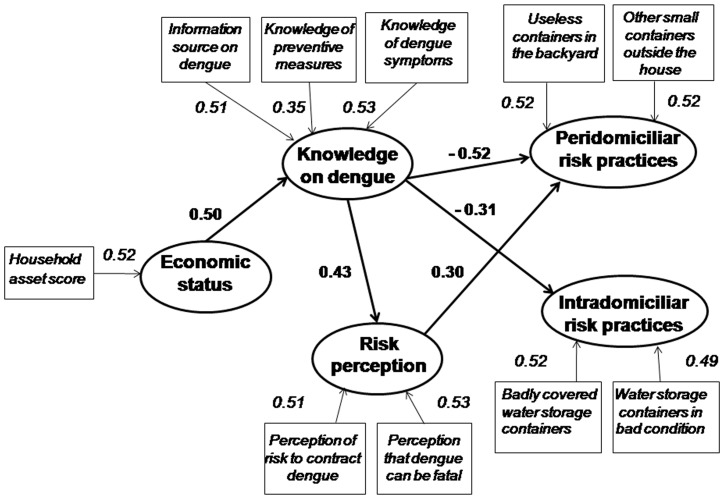
Statistically significant (p<0.05) correlations between Economic status, Knowlegde on dengue, Risk perception and Practices and underlying determinants. La Lisa municipality, Havana City, Cuba.

Figure 2 present the results of the CFA and tests the relationships hypothesized in the underlying conceptual model. The CFA model with the five factors from EFA fitted the data well (χ^2^ = 30.28, *df* = 32; p = 0.553). The Goodness-of-fit was also revealed by high values of the adjusted goodness-of-fit index (0.98).

There were statistically significant correlations between the five factors (latent variables) and all the corresponding observed variables. We confirmed part of the hypothesized direct relationships between factors. Knowledge on dengue had high negative correlations with Intradomiciliar and Peridomiciliar risk practices and a positive correlation one with Risk perception. However, Economic status had a direct significant relationship with Knowledge on dengue only. Although Risk perception in itself was positively related with Peridomiciliary risk practices, it was not related with Intradomiciliry risk.

## Discussion

The CFA model differs from our hypothesized conceptual model, with only Knowledge on dengue having a significant and beneficial direct effect on Practices. However, our results have been obtained with a cross-sectional design and the observed relationships should be interpreted with caution. Still, the study included a large random sample of respondents, and the detailed measurements should strengthen the evidence obtained.

Previous KAP studies on dengue provide conflicting results. Some showed that gaining knowledge does not lead to changing practices [18], [19], [22]. Others provided evidence of a relationship between knowledge of preventive measures and best household practices [4]–[6], [11], [20], [23], [24]. Notwithstanding, studies in Thailand [18], Trinidad and Tobago [19] and Brazil [32] found little or no correlation between knowledge on dengue and *Ae. aegypti* infestation levels. Furthermore, studies presenting evidence on a relationships between knowledge and practices and *Aedes* populations are scarce [11], [13], [23]. It should be noted that there are methodological differences among the cited studies. The main variables were operationalized in various ways. Some authors determined knowledge on dengue as knowledge of symptoms and other disease related aspects such severity or as transmission modes [12], [13], [19], [18], [22], [23], [33]. Other authors included knowledge on the vector breeding sites and a wide range of prevention measures (i.e. covering waters containers, using repellents, and bed nets) [4], [5], [11]. Few others used a score to measure overall dengue knowledge [11], [23]. Something similar is observed in the way actual dengue preventive practices, or their effects, were defined and assessed. Some studies used practices reported by respondents or observed at the time of the study [4], [5], [22], [23], whereas others used information on *Ae. aegypti* larvae, pupal or adult surveys [4], [5], [11], [19], [33]. On the other hand, the methods used to explore the relation between knowledge and practices ranged from calculating association measures to fitting regression models. This should also influence the obtained results.

Perhaps more importantly, associations observed in a particular context may possibly not be extrapolated to other settings. The results of this study suggest a direct association of economic status with dengue knowledge. People with higher economic status could have better access to information through multiple channels (television, radio, newspapers). Combined with the effect of generalized education, this could assure a better understanding and comprehension of information when accessed. On the other hand, economic status was not associated with risk perception and practices.

The way economic status was assessed in this study could be considered a study limitation. Durable material goods owned household level might not reflect the current income of the households. Furthermore, in the Cuban context determinants of health related the economic status, such as access to health services, to social security, to education and to basic food are free or heavily subsidized. Besides, it is difficult to obtain reliable information on real income. However, it is very expensive to acquire durable material goods with an average Cuban monthly salary and supplementary sources of income are needed to do so. Therefore these goods constitute proxy indicators of economic status.

Risk perception was not significantly associated with dengue related practices. This could have several possible explanations. Populatiońs personal experience with the disease could be limited since dengue is not endemic in Cuba and outbreaks occur only from time to time and are rapidly controlled [34]. Besides, communication activities on dengue decrease during inter-epidemic periods. On the other hand, there are contextual cultural factors that influence the practices. In general, there is a technocratic and paternalistic approach to health care. As a result the population does not recognize the importance of its actions in controlling *Ae. aegypti* [35], [36] and has shifted the responsibility to the health sector and other governmental institutions [36].

With regard to increased knowledge and practices for dengue prevention, studies conducted in Santiago de Cuba and Havana [4], [6], [37] showed increased knowledge and adoption of best practices for the reduction of *Ae. aegypti* breeding sites after health education activities. Our study confirms this relationship and suggests that emphasis should be put on concrete ways to prevent dengue.

More generally, we showed that the use of EFA and CFA can clarify the relationship between direct and indirect determinants of dengue related practices and contribute to a better understanding of the potential effect of Information, Education and Communication strategies on the reduction of *Ae. aegypti* breeding sites. In doing so, our study provided inputs for designing a community based strategy for vector control [28], [29] and offers a tool for assessing perceptions and practices related to dengue at household level.
